# Dissecting the Impact of the Gut Microbiome on Cancer Immunotherapy

**DOI:** 10.21203/rs.3.rs-3647386/v1

**Published:** 2023-11-30

**Authors:** Rakesh Jain, Andreas Hadjigeorgiou, Constantinos Harkos, Aditya Mishra, Golnaz Morad, Sarah Johnson, Nadim Ajami, Jennifer Wargo, Lance Munn, Triantafyllos Stylianopoulos

**Affiliations:** Massachusetts General Hospital and Harvard Medical School; University of Cyprus; University of Cyprus; The University of Texas MD Anderson Cancer Center; The University of Texas MD Anderson Cancer Center; The University of Texas MD Anderson Cancer Center; The University of Texas MD Anderson Cancer Center; The University of Texas MD Anderson Cancer Center; Massachusetts General Hospital and Harvard Medical School; University of Cyprus

**Keywords:** mathematical model, microbiome, immunotherapy, immune profiling, probiotics, high-fiber diet, fecal microbiota transplant

## Abstract

The gut microbiome has emerged as a key regulator of response to cancer immunotherapy. However, there is a gap in our understanding of the underlying mechanisms by which the microbiome influences immunotherapy. To this end, we developed a mathematical model based on i) gut microbiome data derived from preclinical studies on melanomas after fecal microbiota transplant, ii) mechanistic modeling of antitumor immune response, and iii) robust association analysis of murine and human microbiome profiles with model-predicted immune profiles. Using our model, we could distill the complexity of these murine and human studies on microbiome modulation in terms of just two model parameters: the activation and killing rate constants of immune cells. We further investigated associations between specific bacterial taxonomies and antitumor immunity and immunotherapy efficacy. This model can guide the design of studies to refine and validate mechanistic links between the microbiome and immune system.

## Introduction

Immune checkpoint blockers (ICBs) have transformed cancer treatment. To date eight different ICBs have been approved alone or in combination with other therapies for ~ 80 indications ^1^. However, less than 20% of patients currently benefit from these treatments ^2^. Moreover, many patients develop immune-related adverse events, some of which can be fatal ^3^. The abnormal and immunosuppressive tumor microenvironment (TME) not only hinders the delivery of ICBs, but also renders them ineffective once they accrue in tumors ^4^. An emerging approach to overcome this challenge is to reprogram the host microbiome ^5,6^.

The impact of the gut microbiome on immunotherapy outcome has been studied across several types of cancers ^7–12^, and an increasing number of clinical and preclinical studies have shown that the diversity, composition, and structure of the gut microbiome is associated with response and resistance to ICB^8,13^. Furthermore, recent experimental trials have demonstrated how fecal microbiota transplants (FMTs) can overcome resistance to ICBs ^13–19^. Despite the strong evidence for the effects of the microbiome on the immune cells and the efficacy of ICBs, little is known about the underlying mechanisms^11^. Limited information and lack of consistency among studies about the positive or negative effects of specific bacteria on ICB efficacy highlight the need to understand how gut microbes affect ICB response.

Mathematical models have enhanced our understanding of not only tumor biology, but also that of other diseases ^20–23^. For instance, mathematical models have been developed for the investigation of interactions among populations of microbes, determining which populations can prevail over others ^24–26^. There are also models focusing on interactions among a small number of bacteria and specific populations of immune cells ^27,28^ or the interactions of the immune system with tumors ^21^. Other models have investigated how interactions between cancer cells and immune cells influence the efficacy of immunotherapy for cancer as well as for cancer patients who have contracted COVID ^22,29^. However, to our knowledge, none of the existing modeling frameworks have analyzed the effect of the microbiome on cancer immunotherapy. Elucidating the role of the microbiome on cancer immunotherapy requires an in-depth investigation of i) the mechanisms by which the microbiome affects activation of cells of both the innate and adaptive immune systems and ii) the positive or negative effects of the various bacterial taxa on immune effector cell function. A fundamental understanding of these processes has the potential to inform new therapeutic strategies. This task is challenging though, as it requires a detailed mathematical framework based on robust experimental data.

**Figure 1:**
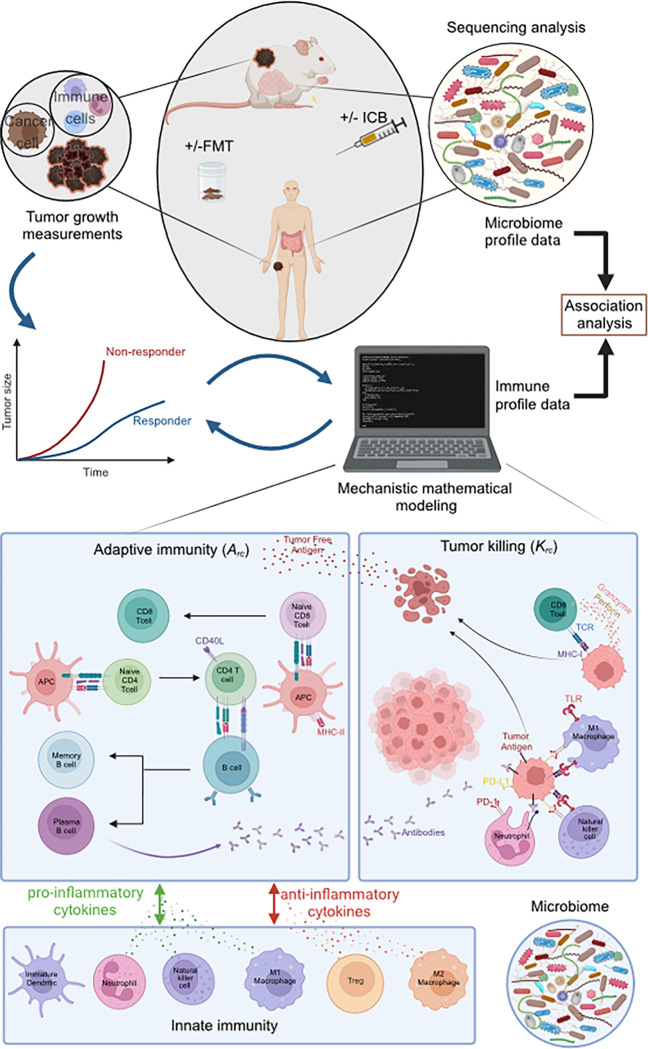
Description of the approach followed in this study to relate immune profiling with microbiome data and schematic of mathematical model components. Our approach involves the reproduction of tumor growth curves with the deterministic mathematical model to set up model parameters and validate model predictions. The tumor growth data as well as the microbiome profiling data are derived from clinical and preclinical studies on melanomas after fecal microbiota transplant and administration of immunotherapy. The validated deterministic model generates the immune profile data for each tumor and the predicted immune profile is associated with the experimentally derived microbiome profiling data. Schematic of mathematical model’s components. The arrows represent the interactions among model components. The intensity of each interaction is associated with the value of a model parameter. Some arrows represent the killing rate constant ($K_{rc}$) and activation rate constant ($A_{rc}$). The model includes the cytolytic effect of CD8+ T cells and Natural killer cells that induce the tumor cells to become antigen. M1 macrophages and immature dendritic cells interact with tumor cells and become antigen-presenting cells (APCs). APCs and Neutrophils interact with tumor cells and antigen inducing phagocytosis. APCs also activate naïve CD4+ and naïve CD8+ T cells to become effector CD4+ and CD8+ T cells, respectively. CD4+ T cells help the activation of Naïve B cells in becoming Plasma and Memory B cells. Plasma cells produce antibodies, which bind to tumor cells. Phagocytosis/apoptosis arrows represent the killing rate constant ($K_{rc}$). The wider the stroke the higher the value of the $K_{rc}$ parameter. Also, some interactions are affected negatively by the binding of PD-1 to anti-PD-L1. Anti-PD-L1and anti-PD-1 reduce the binding of PD1 to PDL1, increasing the intensity of the interaction. In addition, the model incorporates the effects of pro- and anti-inflammatory cytokines and the regulatory effects of M2 macrophages and regulatory T cells (Tregs). Created with BioRender.com

Here we developed a mathematical model of immune checkpoint blockade therapy to investigate the possible mechanisms by which the microbiome influences the immune system. (See Supplementary Information for mathematical equations and parameter values). The model accounts explicitly for interactions among cancer cells, adaptive immunity (i.e., CD4+, CD8 + T cells, regulatory T cells, and B cells), innate immunity (i.e., immature dendritic cells, neutrophils, natural killer cells, and macrophages) and cytokines ^22,29–32^ (see Supplementary Table S1 for a list of the model variables and Figure S1 for the interactions between cells and cytokines). We hypothesize that the gut microbiome affects the immune cells through two distinct processes: their activation and killing efficiency. These processes are incorporated through an activation rate, $A_{rc}$ and a killing rate, $K_{rc}$ (Fig. 1). The values of most model parameters were taken from the literature (Supplementary Table S2). For unknown parameters related to the growth of the tumor and the effects of immunotherapy, the baseline values (Supplementary Table S3) were determined by an optimization procedure to reproduce the average experimental tumor growth curves from relevant mouse studies ^13,14,17^. We then used the model to simulate data from a FMT clinical trial in melanoma patients who progressed on immunotherapy prior to FMT ^16^ and murine models developed to evaluate the impact of responder and non-responder FMT on antitumor response ^17^ for which complete microbiome profiles were available. These simulations yield mechanistic insights about immune cell dynamics in individual subjects. Finally, we performed an association analysis between the immune profiles predicted by our model and the microbiome profile data to discover potential positive and negative dependencies among specific microbial populations and components of the immune system (Fig. 1).

## Results

### Gut microbiome affects the activation and killing potential of immune cells

**Figure 2:**
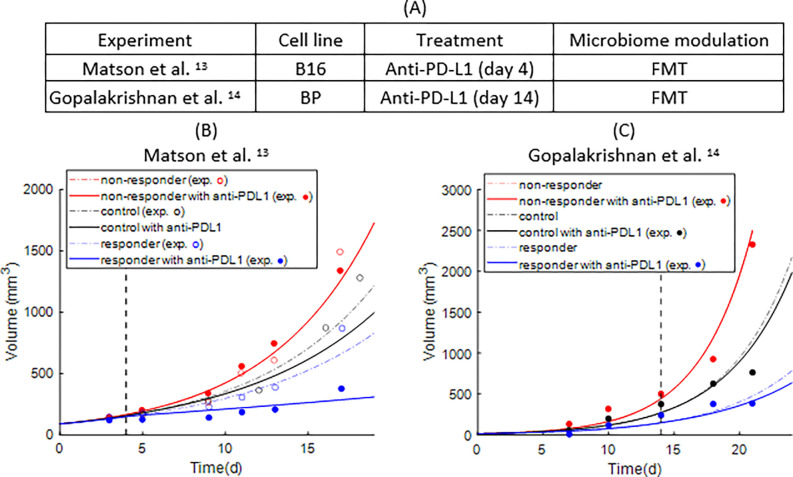
Comparison of experimental tumor growth curves with model prediction to define baseline values of model parameters. A) Experimental details for each experiment. B and C. Bullet points represent the experimental data and the continuous curves represent the model predictions. Day 0 is the initiation of the simulation, B) day 3, C) day 7 of the experiment. The vertical dashed black line represents the initiation of immunotherapy (anti-PD-L1). B) experimental data from Matson et al. ^13^ (B16 melanoma cells) and C) Gopalakrishnan et al. ^14^ (BP melanoma cells. In B and C, the control groups are germ-free mice and the rest of the groups received FMT from either a responder or a non-responder patient to immunotherapy.

We set out to define potential mechanisms by which the gut microbiome affects anti-tumor immune responses. To do so, we first established the values of the model parameters that are involved in the growth of the tumor and the efficacy of immunotherapy (Supplementary Table S3) by fitting the model to experimental data from B16, BP, and HCmel1274 murine melanoma tumors using an optimization precedure^13,14,17^. In the B16 and BP melanoma studies, the gut microbiome was modulated in the case of specific pathogen-free mice or seeded in the case of germ-free mice with a FMT from a responder or a non-responder patient. Subsequently, the mice were treated with anti-PDL1 antibodies (Fig. 2A). The experimental data and model predictions are shown in Fig. 2B–C and Figure S2. For each experiment, we generated a set of values for the model parameters. Among groups of each experiment only the parameter related to the immune cell activation ($A_{rc}$) and the parameter related to the efficiency of cancer cell killing ($K_{rc}$) were varied. Those two parameters describe the hypothesized roles of the microbiome.

**Figure 3:**
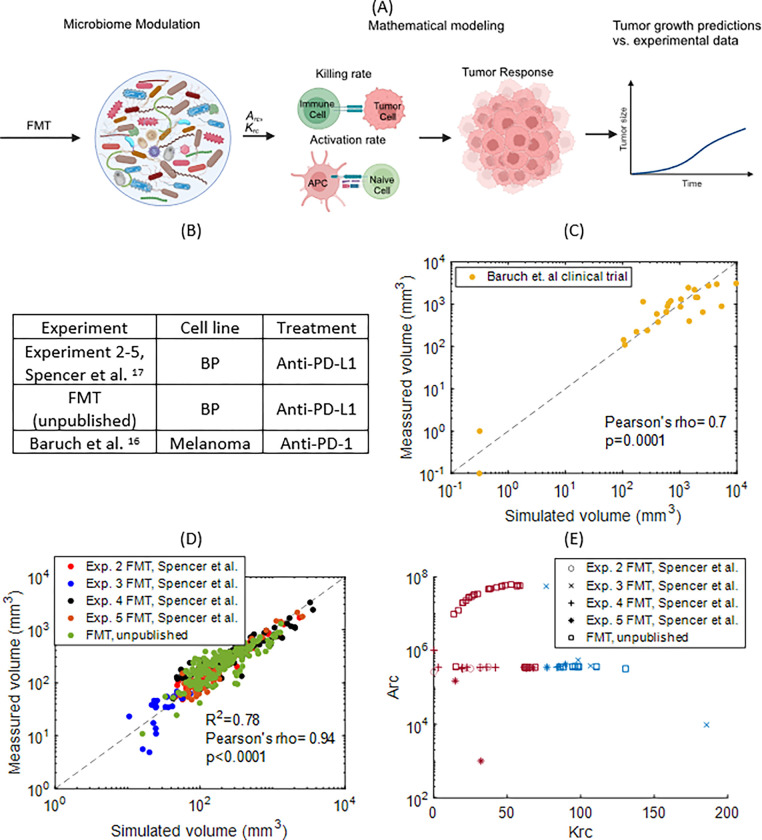
Model validation with preclinical and clinical data. Microbiome modulation was assumed to affect only the parameters $K_{rc}$ and $A_{rc}$. Comparison of model predictions with measured. A) Schematic of followed procedure (Created with BioRender.com) B) Experimental details for each experiment C) clinical data that include 9 patients (Baruch et al. ^16^) and D) preclinical tumor volume data that include 62 mice which received only FMT modulation of microbiome (Spencer et al. ^17^ and an unpublished FMT data set). E) Range of $K_{rc}$ and $A_{rc}$ parameters employed for the results in figure 3D, blue and red points correspond to responders and non-responders, respectively.

Next, we employed data from a clinical study of patients with melanoma who progressed on ICB and were subsequently treated with FMT followed by ICB in an attempt to overcome initial ICB resistance (Baruch et al.^16^). Here, we assigned the same baseline parameter values obtained from the HCmel1274 murine melanoma study (Spencer et al. ^17^ Supplementary Figure S2) because these studies used the same anti-PD1 treatment; we then simulated treatment of the human melanoma tumors (Fig. 3A–C). To reproduce these data, we only varied the parameters related to the immune cell activation ($A_{rc}$) and efficiency of cancer cell killing ($K_{rc}$) which we assumed are affected by microbiome (as shown in Fig. 3A). The model was able to reproduce the clinical tumor growth data with good accuracy (Pearson’s rho, r = 0.7 with a p-value 0.0001) as well as the data for CD8 + T cells (Supplementary Figure S3).

After defining baseline values of the model parameters, we then used the model to probe new sets of pertinent experimental data for which complete sets of microbiome profiles were available. To reproduce these data, we only varied the parameters $A_{rc}$ and $K_{rc}$. Specifically, we used data from 62 mice (Fig. 3D) where: i) 35 received FMT from a responder, a non-responder melanoma patient, or a healthy person (FMT, unpublished data) and ii) 27 received FMT from a responder patient (experiments 2–5 from Spencer et al. ^17^). For experiments 2–5 and FMT, the baseline parameters were kept the same as for Gopalakrishnan et al. ^14^ (Fig. 2C and Supplementary Table S3) because both of these experiments involved the same cell line (BP melanoma) and the same treatment (anti-PD-L1).

This process resulted in fits for the killing rate constant (parameter $K_{rc}$) and the activation of the adaptive immune system (parameter $A_{rc}$) for each mouse. Figure 3D presents the comparison of the experimental and the predicted tumor volume by the model at all available time points. The diagonal dashed line depicts the best-fitting case scenario. Points close to the diagonal line have the best agreement between the simulated and experimental values. By only varying $K_{rc}$ and $A_{rc}$ and keeping the rest of the parameters at their baseline values, the model was able to reproduce the measured tumor volume for all 62 mice with good accuracy (R^2^ = 0.78; Pearson’s rho, r = 0.94 with a p-value less than 0.0001). This supports our hypothesis that the primary effects of the microbiome are through modulation of the killing of tumor cells by immune cells and the activation of the adaptive immune system. Figure 3E shows the range of values of $K_{rc}$ and $A_{rc}$ that correspond to responders and non-responders (blue and red points respectively). A tumor was assumed to be a responder if its simulated volume decreased below a threshold value (10 mm^3^) and never surpassed a size of 1500 mm^3^ until day 100.

### Modeling framework suggests antitumor immune responses

**Figure 4:**
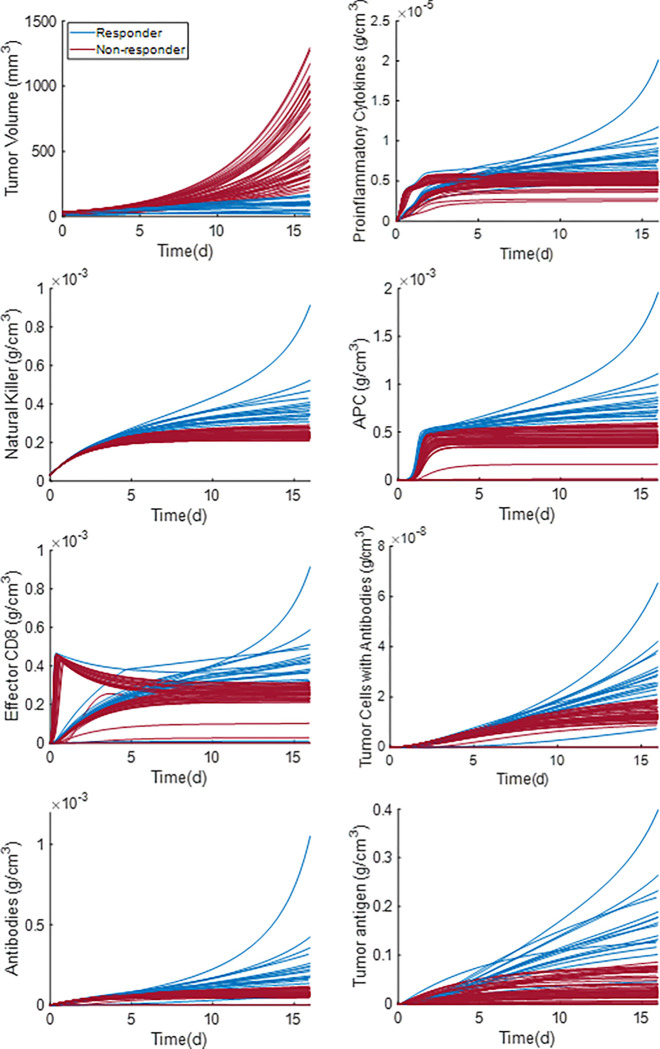
The time evolution of indicative model variables. The blue curves represent the variables for the responder group and the red curves for the non-responders respectively.

The advantage of mathematical modeling is the ability to estimate mechanistic parameters that are difficult to determine experimentally. From the analysis in Fig. 3D, our model predicted the immune profile of the 62 cases for which the microbiome profile was available. Predictions of model variables for the antitumor immune response are shown in Fig. 4. The figure presents the time evolution of model variables, and the plots are divided into two categories representing the tumor response – responders and non-responders. A tumor was assumed to be a responder if its simulated volume decreased below a threshold value (10 mm^3^) and never surpassed a size of 1500 mm^3^ until day 100. The non-responder concentrations (red curves) of pro-inflammatory cytokines, natural killer cells, and antigen-presenting cells have lower values than the corresponding concentrations for the responders (blue curves), and they reach a plateau. The activation of the adaptive immune system is also insignificant in the non-responders and thus, the non-responder concentrations of the effector CD8 + and CD4 + T cells, memory, and plasma B cells are near zero, which results in a low amount of tumor antigen and tumor cells with antibodies. On the other hand, the responders’ concentrations of the pro-inflammatory cytokines, natural killer cells, and antigen-presenting cells are continuously increasing. There is also a significant activation of the adaptive immune system in responders, which results in high concentrations of effector CD8 + and CD4 + T cells, memory, and plasma B cells. Overall, the simulations predict that the responder mice have strong innate and adaptive immune responses, which results in decreased tumor cell density and tumor eradication.

### Association analysis suggests correlations of specific gut bacteria with immune cell responses

**Figure 5:**
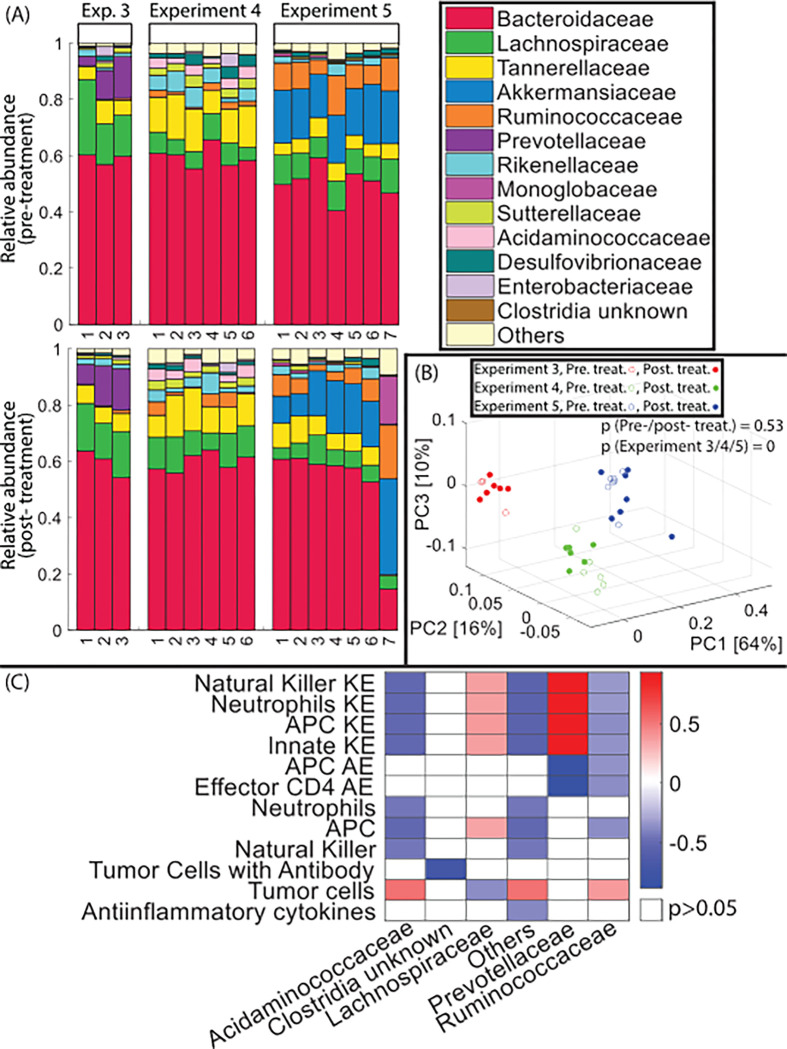
Microbial abundance profiles obtained by 16Sv4 RNA gene profiling of murine fecal samples. (A) Fecal microbiome profiles are represented by compositional plots showing the relative abundance of the bacteria at the family level. the three experimental groups (E1, E2, and E3) received only FMT microbiome modulation and treated with a-PD-L1 (B) Principal components analysis of the microbiome data and the p-value of PERMANOVA for pre or post treatment groups and the experimental groups. C) The association between microbiome at the family level and immune profile by adjusting for the three experiments (only values with p<0.05 are presented).

Next, we examined the relationships between specific microbes and components of the immune system. To this end, we performed a robust association analysis between the model predictions of the immune system with the microbiome profiling (Fig. 5A,B) from the murine studies (experiment 3 4 and 5 in Fig. 3)^17^ of mice that received microbiome modulation only with FMT. To identify associations of the microbiome families with components of the immune system, we evaluated the association of microbiome profiles reported in the experimental studies with the immune profiles generated by our model. For the association analysis (Fig. 5C), the microbiome profiling data (Fig. 5A) were not separated to pre- and post-ICB treatment because the underlying microbiome profiles are not significantly different as seen in Fig. 5B for the p-value of pre-/post-treatment. The three different experiments involve studies with three different FMT responder donors. The different FMT donors induce different microbiomes, as evidenced by the microbiome profiles in Figs. 5A and B (p-value of the experiment 3,4, and 5 is 0). The association analysis is presented in Fig. 5C and summarized in Table 1. We have suitably adjusted for the three experiments by only considering the microbiome families that have non-zero relative abundance for their associations. In this way, we have the largest possible data set that allowed us to extract the strongest signal from all experiments.

Our model identified certain bacterial taxa that have been the subject of previous studies, such as Lachnospiraceae, Prevotellaceae and Ruminococcaceae ^33–36^. The analysis Fig. 5C suggests that there are direct associations between the immune cell killing and activation efficiencies ($K_{rc}$ and $A_{rc}$ respectively in Fig. 5C) and the bacterial taxa. These associations are consistent with our initial hypothesis that the microbiome affects the immune system primarily by enhancing immune cell activation and killing efficiency. The results also show that Lachnospiraceae is correlated positively with the killing effectiveness of the innate immune cells (natural killer, neutrophils and APCs) and negatively with the number of tumor cells. On the other hand, the Acidaminococcaceae and “Other” families (families that had a very small relative abundance) are correlated negatively with the killing effectiveness of the innate immune cells and positively with tumor cells. The Ruminococcaceae family is correlated negatively with the killing effectiveness of the innate immune cells and activation effectiveness of the adaptive immune cells, which results in a positive correlation with tumor cells. Interestingly, the Prevotellaceae family seems to have a dual role: it is correlated positively with the killing effectiveness of the innate immune cells and negatively with the activation effectiveness of the adaptive immune cells.

## Discussion

In this study, we developed a systems approach to elucidate the role of the microbiome in controlling immune responses and determining the efficacy of immunotherapy. Our approach is based on (a) microbiome profiles obtained from preclinical and clinical studies, (b) a mechanistic mathematical model specifically developed for the simulation of the immune profile during anti-tumor response and (c) an advanced statistical analysis to correlate the experimental data with the model predictions. Through comparison of model predictions with experimental observation of tumor response, we identified the immune activation rate, $A_{rc}$, and the killing rate, $K_{rc}$, as key determinants of microbiome-mediated anti-tumor immunity. Provocatively, using our mathematical model, we were able to distill the complexity of each experiment and microbiome modulation with just two model parameters: the killing rate constant ($K_{rc}$) and the activation rate constant ($A_{rc}$) (Fig. 3). Furthermore, by evaluating the association of different bacterial taxa with the immune profiles predicted by our model, we identified patterns of association that provide additional information about the involvement of the microbiome in the response and resistance to ICB. These results can inform future pre-clinical and clinical studies. Interestingly, the microbiome association analysis indicates direct connections between the microbiome and killing and activation efficiency of the adaptive immune cells. Our results can also help guide experimental investigations of these two crucial mechanisms and how they are affected by specific microbiome families. Of note, our model and methodology can be used as a tool to test and refine new mechanisms as soon as new experiments become available.

Regarding the association analysis of the microbiome families and the immune components, the Lachnospiraceae family is correlated positively with increased immune responses, which is in agreement with the literature ^33,34^. The Ruminococcaceae family seems to induce immunosuppression according to our results which is consistent with literature reports that this family has anti-inflammation effects on the colon and causes less adverse effects during immunotherapy ^35–37^. In patients with melanoma, the Ruminococcaceae family was mostly found in responders to immunotherapy ^14^. This discrepancy indicates that the microbiome has a collective effect on the response to immunotherapy and a balance between immunostimulation to cause anti-tumor effects and immunosuppression to limit adverse effects is needed to induce response to immunotherapy.

It should be noted that the analyses based on our model do not imply causal relationships between bacteria and the tumor immune profile. Nevertheless, the identified associations with immune components could help determine the focus and design of future mechanistic biological studies. In addition, as with any mathematical model, our modeling framework is subject to certain assumptions and limitations. The most obvious limitations are that we focused on the gut microbiome and did not consider the local tumor microenvironment. Also, we assumed that the contribution of the microbiome to the activation and killing rates are the same for all cells of the innate and adaptive immune systems. Furthermore we did not consider in the deterministic model the role of PD-L2 ligand ^38^. Due to the complexity of the immune system, it would be intractable for all individual components to be incorporated into a model with parameters that can be estimated independently. These limitations not withstanding, our systems approach combining state-of-the-art murine and human studies with advanced mathematical modeling and statistical analysis paves the way for the elucidation of the role of microbiome modulation in improving cancer immunotherapy.

## Methods

### Mathematical model description

The mathematical model consists of a set of ordinary differential and algebraic equations that are solved in MATLAB. Each model variable represents a model component. The components are: immune cells, tumor cells, cytokines and antibodies, including anti-PD-L1 and anti-PD-1 antibodies (Fig. 1). The model considers the effect of pro- and anti-inflammatory cytokines on immune cell functions, the activation of naïve immune cells, and the killing of cancer cells by immune cells. The model also includes cancer cells in three different states: alive cancer cells, dead cancer cells that released free tumor antigen, and cancer cells targeted with antibodies. In addition, we account for the binding of PD1 to PDL1 on the immune cell surface, which reduces their killing or activating potential. Injection of anti-PD-L1 or anti-PD-1 immunotherapy blocks PD-L1 or PD-1, respectively, on the cells enhancing the killing efficacy and activation efficacy of the adaptive immune system. The killing rate constant ($K_{rc}$) represents the effectiveness of the tumor cells’ killing by the immune cells. As the killing potential of cells of the adaptive immune system is considered more robust compared to that of the innate system, a higher value of $K_{rc}$ is assigned to the cells of the adaptive immune system. The two $K_{rc}$ values are related with a proportionality constant. This is done to account for the more efficient killing of tumor cells by CD8 effector T cells, as shown in Fig. 1. The activation rate constant ($A_{rc}$) represents how easily a naïve cell can be converted into an effector cell. We assumed that the microbiome affects the immune response through these two parameters, $K_{rc}$ and $A_{rc}$. A detailed description of the model equations is provided in Supplementary Information.

The growing tumor was simulated by assuming that all interactions take place inside the tumor region. Furthermore, a uniform spatial distribution was assumed initially for the values of the model variables. The density of the tumor region that includes the immune cells and tumor cells was assumed constant through time and equal to the initial cancer cell density. Based on these assumptions and by calculating the reaction rates of all model components, the rate of change of tumor volume was evaluated. The mass balance for each component of the model was solved and the tumor volume was calculated from the total mass of cells that comprise the tumor (details in Supplementary Information)

The values of many model parameters were taken from the literature (Supplementary Table S2). To determine the values of the remaining parameters, we employed an optimization procedure to reproduce experimental tumor growth curves from published studies ^13,14,17,39^ by varying these parameters. The optimization function was the minimization of the sum of the squared difference of the logarithmic values between the measured ($V_{\exp\text{.}}$) and simulated ($V_{\mathrm{calc}\text{.}}$) tumor volumes $\left(min\left(\sum_{i}{\left[ln\left(V_{exp,i}\right)-ln\left(V_{\mathrm{calc},i}\right)\right]}^{2}\right)\right)$. Only positive values of model parameters were accepted by the optimization algorithm to have a physical meaning. This was performed by introducing a penalty into the optimization function for negative parameters. The Nelder–Mead simplex algorithm ^40,41^ was used to find the minimum of the optimization function. The baseline model parameters that depend on tumor cell type and immunotherapy type (anti-PD-L1 and anti-PD-1) are defined with the optimization algorithm by using the average experimental volume from Matson et al. ^13^, Gopalakrishnan et al. ^14^ and Spencer et al. ^17^ for B16, BP and HCmel1274 melanoma cells. The baseline parameters for each combination of tumor cell/immunotherapy type were kept the same for the validation of tumor growth curves for each individual subject with the same tumor cell and immunotherapy type, and only the parameters $K_{rc}$ and $A_{rc}$ were varied to reproduce the other experimental datasets. The aforementioned process assessed the predictive capabilities of the model by varying only two parameters that affect crucial mechanisms of the immune system.

Using the observed tumor volume data, we trained our mathematical model to obtain an optimal estimate of $K_{rc}$ and $A_{rc}$. We used the forward model parameters estimate to learn the state of the immune system in terms of the concentration of the various types of immune cells and cytokines. Recent advances in immunotherapy have shown that the microbiome mediates through the immune system to affect tumor growth in cancer patients ^12–19^. We have accessed the overall significance of some of the observed confounders, such as experiment type (E1, E2, and E3) and treatment (pre/post-PD-L1), with PERMANOVA (Fig. 5B). The analysis shows that experiment types significantly alter microbial diversity. Accordingly, we compute the association between the microbiome and the predicted immune components with Spearman’s Rank correlation analysis.

### Description of murine and human studies used for validation of the model

#### Murine studies

The experimental design for these studies has been previously described in full detail ^17^. The studies were conducted at the MD Anderson Cancer Center under the approval of the Institutional Animal Care and Use Committee (IACUC). B6 germ-free mice were colonized by a complete responder microbiota through FMT. One week was allowed for engraftment, following which, mice received subcutaneous injection of BP melanoma cells. Once tumors reached a size range of 250–500 mm^3^, mice were treated with 3 doses of intraperitoneal anti-PD-L1 and tumor size was measured using a caliper.

#### Human studies

For further validation of our model, we incorporated data from a phase 1 clinical trial conducted by Baruch et al. ^16^ that evaluated the safety of FMT in patients with PD-1-refractory melanoma. Ten patients were enrolled in this study and received a broad-spectrum antibiotic treatment to deplete the native microbiota, followed by FMT with stool samples from complete responders (2 donors). FMT was conducted via colonoscopy and continued via oral capsules at day 1 and day 12 post-colonoscopy. Subsequently, patients received anti-PD-1 treatment with additional FMT (oral capsules) administered every 14 days. Response to tumor treatment was measured using the iRECIST criteria.

### Statistical analysis

We used statistical methods to study the connection between the microbiome and the immune system in experiments 3 4 and 5 ^17^. These experiments involved changing the microbiome only through FMT. We used a beta-diversity analysis to compare diversity between samples and represented each sample using the top three principal components of principal coordinate analysis. We also analyzed the significance of observed confounders and ICB treatment on microbiome using PERMANOVA analyses. This helped us identify confounders that had a significant impact on the diversity of the microbiome. After controlling these confounders, we measured the association using Spearman’s Rank correlation. We used the R statistical software and MATLAB for all analyses and visual representations.

## Figures and Tables

**Table 1:** Summary of the association between microbiome at the family level and immune profile for the fiber experiment. Table shows the association between microbiome and adaptive-innate immunity before and after treatment.

	Post-treatment	
	Adaptive	Innate
Acidaminococcaceae	↓	-
Clostridia unknown	↓	–
Lachnospiraceae	-	↑
Others		↓
Prevotellaceae	↓	↑
Ruminococcaceae	↓	↓

Positive association ↑ Negative association ↓ No indication –

## Data Availability

Data can be retrieved from the corresponding authors upon reasonable request.
